# Diffusion Tensor Imaging Along the Perivascular Space Is a Promising Imaging Method in Parkinson's Disease: A Systematic Review and Meta‐Analysis Study

**DOI:** 10.1111/cns.70434

**Published:** 2025-05-16

**Authors:** Kiarash Shirbandi, Mostafa Jafari, Fatemeh Mazaheri, Marziyeh Tahmasbi

**Affiliations:** ^1^ Department of Biomedical Engineering Faculty of Medical Sciences and Technologies, Science and Research Branch, Islamic Azad University Tehran Iran; ^2^ Department of Radiologic Technology, School of Allied Medical Sciences Ahvaz Jundishapur University of Medical Sciences Ahvaz Iran; ^3^ Department of Medical Physics and Biomedical Engineering School of Medicine, Tehran University of Medical Sciences (TUMS) Tehran Iran; ^4^ Department of Radiologic Technology School of Allied Medical Sciences, Ahvaz Jundishapur University of Medical Sciences Ahvaz Iran

**Keywords:** ALPS index, diffusion tensor imaging, glymphatic function, Parkinson's disease

## Abstract

**Introduction:**

Parkinson's disease (PD) is a chronic, progressive neurodegenerative disorder that primarily affects motor functions. Recently, a diffusion tensor imaging technique called DTI along the perivascular space (DTI‐ALPS) has gained attention as a noninvasive biomarker for glymphatic function. This systematic review and meta‐analysis aimed to evaluate the potential and implications of the DTI‐ALPS index for diagnosing PD.

**Methods:**

This study followed the PRISMA 2020 statement. Eligible cohort and cross‐sectional studies measured the ALPS index in PD patients versus non‐PD participants. Web of Science, Medline, Scopus, Embase, Cochrane, PROSPERO, and ICTRP databases were explored until November 14, 2024. Two researchers independently screened studies, extracted data, and assessed the risk of bias using the Newcastle–Ottawa Scale (NOS). The meta‐analysis used a random effects model (REM), assessing heterogeneity (*I* ^2^, Q‐test) and publication bias (Egger's test, trim&fill plot). The certainty of the evidence was evaluated using the GRADE approach.

**Results:**

This meta‐analysis of 11 studies, involving 1462 patients (855 PD, 607 non‐PD of both genders), yielded significant findings. The overall ALPS index differed substantially between PD and non‐PD groups (SMD: −0.61, 95% CI: −0.72, −0.50, *p* < 0.001). Additionally, a significant negative correlation emerged between the ALPS index and Unified PD Rating Scale III (UPDRS III) (r = −0.40, (95% CI: −0.59, −0.18, *I* ^2^: 89.81, *p* < 0.001)), indicating glymphatic dysfunction's impact on cognitive decline. However, a weak and statistically non‐significant correlation was observed between the ALPS index and Montreal Cognitive Assessment (MoCA) (*r* = 0.24, 95% CI: −0.32 to 0.68), with high heterogeneity across studies (*I* ^2^ = 87.37, *p* < 0.001 for heterogeneity). Publication bias risk was low for the overall ALPS index.

**Conclusion:**

These findings highlight the potential of DTI‐ALPS as a noninvasive biomarker for PD diagnosis and progression monitoring. Further studies are warranted to explore its applicability in differentiating PD from other neurodegenerative disorders.

## Graphical abstract

This meta‐analysis of 11 studies (*n* = 1462) demonstrates that the DTI‐ALPS index is significantly lower in Parkinson's disease (PD) patients compared to healthy controls (SMD: −0.61, *p* < 0.001), correlating with motor severity (UPDRS III: *r* = −0.40). These findings support DTI‐ALPS as a noninvasive biomarker for glymphatic dysfunction in PD.

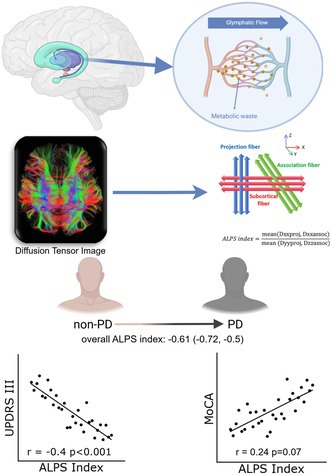

## 1 Introduction

Parkinson's disease (PD) is a chronic, progressive neurodegenerative disorder that primarily affects motor functions, such as bradykinesia, resting tremors, and rigidity. However, it also manifests with a range of non‐motor symptoms, including cognitive impairment, sleep disturbances, and autonomic dysfunction, which collectively diminish the quality of life for patients as the disease progresses [1, 2].

The degeneration of dopaminergic neurons has long been recognized as a central feature of PD. In addition to dopaminergic neuron degeneration, emerging evidence suggests that dysfunction in the brain's waste clearance system, known as the glymphatic system, may also contribute to PD pathology [3, 4]. The glymphatic system clears metabolic waste and neurotoxic proteins from the brain, such as alpha‐synuclein (α‐syn) and tau. These proteins, particularly α‐syn, are central to PD pathology, accumulating in neuronal tissues and contributing to neurodegeneration [1, 4]. Functioning through perivascular channels, the glymphatic system facilitates the exchange of cerebrospinal fluid (CSF) and interstitial fluid, aiding in removing toxic proteins from the brain. Dysfunction in this clearance system has been linked not only to PD but also to other neurodegenerative diseases, such as Alzheimer's disease (AD), underscoring its broad significance in neurological health [3].

In recent years, a technique called diffusion tensor imaging (DTI) along the perivascular space (better known as the DTI‐ALPS index) has emerged as a non‐invasive neuroimaging biomarker capable of assessing glymphatic function [2]. The DTI‐ALPS index is a diffusion imaging‐derived metric designed to assess glymphatic function by quantifying water diffusivity along perivascular spaces (PVS) [5]. This index focuses on specific regions of interest, such as the projection fibers and association fibers, which are believed to reflect the activity of the glymphatic system [6]. By evaluating anisotropic diffusion in these areas, the DTI‐ALPS index provides insights into the waste clearance capacity of the brain [5]. A high ALPS index indicates efficient fluid movement along PVS, correlating with healthy glymphatic function, while a lower ALPS index suggests impaired clearance, as observed in neurodegenerative disorders like PD [7]. This method offers a non‐invasive approach to studying glymphatic dysfunction and its role in disease pathophysiology, making it a promising biomarker for early diagnosis and progression monitoring. This imaging tool has demonstrated its potential in correlating glymphatic dysfunction with motor and cognitive symptoms in neurodegenerative diseases, making it a promising marker for understanding disease progression [5]. Specifically, Jung Bae et al. [8] and Qin et al. [9] applied this technique to detect PD, highlighting its utility in identifying early glymphatic changes associated with the disease. Additionally, Steward et al. [10] and Kamagata et al. [11] employed the DTI‐ALPS index to study mild cognitive impairment (MCI) and Alzheimer's disease, further demonstrating its versatility in assessing glymphatic function across various neurodegenerative conditions.

Given the growing evidence supporting the role of glymphatic dysfunction in PD and the potential of the DTI‐ALPS index to assess this dysfunction, this study systematically evaluates its utility as a biomarker for PD detection. While individual studies have explored this index, a comprehensive meta‐analysis is necessary to quantify its diagnostic value across diverse clinical settings, address variability in research methodology, and refine the understanding of its role in PD pathophysiology. Through this systematic review and meta‐analysis, we integrate evidence to assess the index's diagnostic and clinical significance, emphasizing its potential for early diagnosis and disease monitoring. Additionally, this study addresses methodological limitations and research variability, interpreting the DTI‐ALPS index in the context of glymphatic function. By consolidating current knowledge and identifying research gaps, our findings lay the foundation for advancing the clinical application of the DTI‐ALPS index, improving diagnostic accuracy, monitoring disease progression, and refining therapeutic strategies to enhance patient outcomes in PD.

## 2 Methods

This study follows the Preferred Reporting Items for Systematic Reviews and Meta‐Analyses (PRISMA) statement 2020 [12] and is registered in the International Prospective Register of Systematic Reviews (PROSPERO) (*#CRD42024586834*). Overall, this study did not deviate from the submitted protocol. The right and left sides of the ALPS index were not included in this analysis due to limitations in the number of studies reporting these separately. The exclusion of lateralized ALPS index data may limit the ability to assess hemisphere‐specific glymphatic dysfunction. Instead, the analysis utilized the overall average ALPS index value when reported, as it represents a more comprehensive measure of global glymphatic function while minimizing variability associated with lateralization effects. Additionally, both cohort and cross‐sectional studies were included in the analysis. This decision was made because cross‐sectional designs were predominant among previous studies, and case–control designs were not commonly used.

### 2.1 Eligibility Criteria

All cohort and cross‐sectional studies that measured the ALPS index retrospectively or prospectively in patients with PD were included in this study. Only human subjects' studies (including both male and female genders) that compared the PD with non‐PD participants were considered. All studies included participants (≥ 18 years) were eligible by clinical evaluation tools such as the Unified Parkinson's Disease Rating Scale III (UPDRS‐III), Hoehn and Yahr (H‐Y) rating scale, and Montreal Cognitive Assessment (MoCA) for assessing motor disability. Studies with a single group, PD with other cognitive symptoms, or neurological diseases were excluded from this study.

### 2.2 Information Sources

The primary search was done through Web of Science (WoS), Medline, Scopus, Embase, Cochrane, and PROSPERO databases. The trials were searched among ClinicalTrials.gov and ICTRP databases. Moreover, conference papers, gray literature, and unpublished articles were searched in AllConferences, ConferenceAlerts, OATD, and OpenGrey as gray literature databases. The search included articles published in English up to October 20, 2024, without restrictions on study design. Additionally, the backward and forward citation tracking method was applied to ensure comprehensive coverage of relevant articles.

### 2.3 Search Strategy

All relevant Medical Subject Headings (MeSH) and non‐MeSH terms were established to find the articles from the databases (#1 “Parkinson's Disease” OR “Brain Diseases” OR “Basal Ganglia Diseases” OR “Parkinsonian Disorders”; #2 “Diagnostic Imaging Tomography” OR “Magnetic Resonance Imaging” OR “Diffusion Tensor Imaging” OR “analysis along the perivascular space” OR “ALPS” or “DTI‐ALPS”; #3 “Central Nervous System” OR “Brain” OR “Glymphatic System”) (Data S1).

### 2.4 Selection Process

The selection process was conducted independently by two researchers (M.J. and F.M.), who were blinded to each other's results. They performed an initial search and screening of titles and abstracts, followed by a full‐text evaluation to identify relevant studies. After completing their independent assessments, discrepancies were resolved through discussion. If consensus could not be achieved, a third researcher (K.Sh.) provided the final decision. The initial screening was facilitated using the Rayyan tool [13], and the findings were subsequently imported into EndNote X9 software to identify and manage duplicates.

### 2.5 Data Collection Process

Data were extracted independently by three researchers (M.J., F.M., and M.T.) within a standardized checklist. In cases of unresolved discrepancies, a fourth researcher (K.Sh.) made the final decision. The mean ± standard deviation (SD) of ALPS indices was directly obtained from the articles when available. For extraction data from figures without reported data, GetData Graph Digitizer (Version 2.26) was employed. Prior to indirect data extraction, the researchers attempted to contact the authors via email three times to request the necessary data.

### 2.6 Data Items

A standardized form was utilized by three researchers (M.J., F.M., and M.T.) to extract data from the included studies. The extracted information encompassed various aspects, including study characteristics (author, location, year, study design, type of center, and primary or secondary analysis), population features (age, gender, and PD or non‐PD) and group sizes, main outcomes, limitations, key results, and specific data points such as DTI‐ALPS values. For the prognosis meta‐analysis, the following data were extracted: mean ± SD for the overall ALPS index and Pearson correlation coefficients between the overall ALPS index, UPDRS‐III, and MoCA. To assess the relationship between the ALPS index and clinical measures (UPDRS‐III and MoCA scores), we initially extracted Pearson correlation coefficients from the included studies. However, to facilitate a standardized meta‐analysis and ensure comparability across studies with different effect size metrics, we converted the correlation coefficients into standardized mean differences (SMD) using established statistical transformation methods. This approach allows for a consistent effect size representation while maintaining statistical robustness. Although SMD provides a comparable measure of association, we acknowledge that directly reporting correlation coefficients may offer additional interpretability. Future studies should consider direct correlation meta‐analysis to enhance precision in evaluating these relationships. This comprehensive data extraction process enabled a thorough analysis of the included studies. However, we acknowledge the limitations associated with a small sample size (*n* = 11), including potential sensitivity to deviations from normality and outliers.

### 2.7 Study Risk of Bias Assessment

The Newcastle–Ottawa Scale (NOS) was employed to evaluate individual study bias, ensuring rigorous quality assessment. The NOS checklist assesses three key domains: selection (participant selection and recruitment), comparability (control for confounding variables), and outcome (cohort studies) or exposure (case–control studies).

Studies were then rated based on their total score: high quality (greater than 7), medium quality (5 to 6), and low quality (less than 5). This assessment helped identify potential biases and evaluate the overall quality of the studies, with details provided in the Supporting Information.

### 2.8 Effect Measures

A meta‐analysis was performed to evaluate the overall ALPS index, and the mean difference (MD) with 95% confidence intervals (CI) and standardized mean difference (SMD) (95% CI) were used to compare the ALPS index between PD and non‐PD [14].

Pearson's correlation coefficients (r) were converted to Fisher's z‐scores [15, 16, 17] using the formula:

$$Z_{r}=\frac{\ln\left(\left(1+r\right)/\left(1-r\right)\right)}{2}$$

To stabilize the variance and approximate a normal distribution, which is necessary for valid meta‐analytic comparisons across studies, the standard error of Fisher's z was calculated as:

$$SE_{Z_{r}}=\frac{1}{\sqrt{n-3}}$$

where *n* is the sample size. This transformation ensures the compatibility of effect sizes for pooling and weighted analyses in the generic method [16].

### 2.9 Synthesis Methods

Stata MP (V.17.0) with the “metan” package (StataCorp, College Station, Texas, USA) was used for inclusion studies with a significance level of *p*‐value < 0.05. The SMD effect size was measured by Hedges's g index [18]. The effect size was categorized into four distinct levels to facilitate interpretation. No effect was indicated by values less than −0.2, while small, medium, and large effects were represented by ranges of 0.2–0.5, 0.5–0.8, and greater than 0.8, respectively. This classification enabled a clearer understanding of the study's findings and their practical implications [19]. Based on the methodologic heterogeneity, the random effects model (REM) with restricted maximum likelihood (REML) was selected [20]. Moreover, for comparing between groups that were under 10 studies, the Hartung–Knapp modification methods with prediction interval (95% CI) were used [21].

Heterogeneity was assessed using *I* ^2^ and Q‐test (χ^2^) statistics, with a significance level of ≤ 0.05. The degree of heterogeneity was classified into four levels: low (0–40%), indicating negligible impact; moderate (30%–60%), suggesting potential influence; substantial (50%–90%), signifying significant impact; and considerable (75%–100%), indicating pronounced differences [22].

Subgroup analysis was performed to identify potential sources of heterogeneity for results with *I* ^2^ upper than 50% [23], and sensitivity analysis was based on five strategies: (1) the leave‐one‐out remove method, (2) subgroup based on the NOS quality assessment score, (3) type of centers, (4) study designs, and (5) primary or secondary analysis to assess the impact of individual studies on the overall results.

### 2.10 Reporting Bias Assessment

Egger's test and plot (with a significance level of *p* ≤ 0.10), along with the trim & fill method, were used to assess small‐study effects (publication bias) when the number of included studies was fewer than 10 [24, 25, 26]. Moreover, contour funnel plots and Doi plots were reported for analysis with at least 10 studies. The Doi plot was assessed by LFK index with the following categories: no asymmetry (−1 < LFK < 1), minor asymmetry (LFK −2 to −1/1 to 2), and major asymmetry (< −2/ > 2) [27].

### 2.11 Certainty Assessment

The certainty of evidence using the Grading of Recommendations, Assessment, Development, and Evaluation (GRADE) approach was assessed [28]. The GRADE criteria considered study limitations (risk of bias and methodological quality), inconsistency (heterogeneity between studies), indirectness (relevance of study population, intervention, and outcomes), imprecision (uncertainty due to sample size and effect size), and publication bias (potential for selective reporting). The certainty levels were categorized as high (confident in effect estimate), moderate (likely true but uncertain), low (uncertain with potential bias), and very low (extremely uncertain with a high risk of bias).

## 3 Results

### 3.1 Study Selection

**FIGURE 1:**
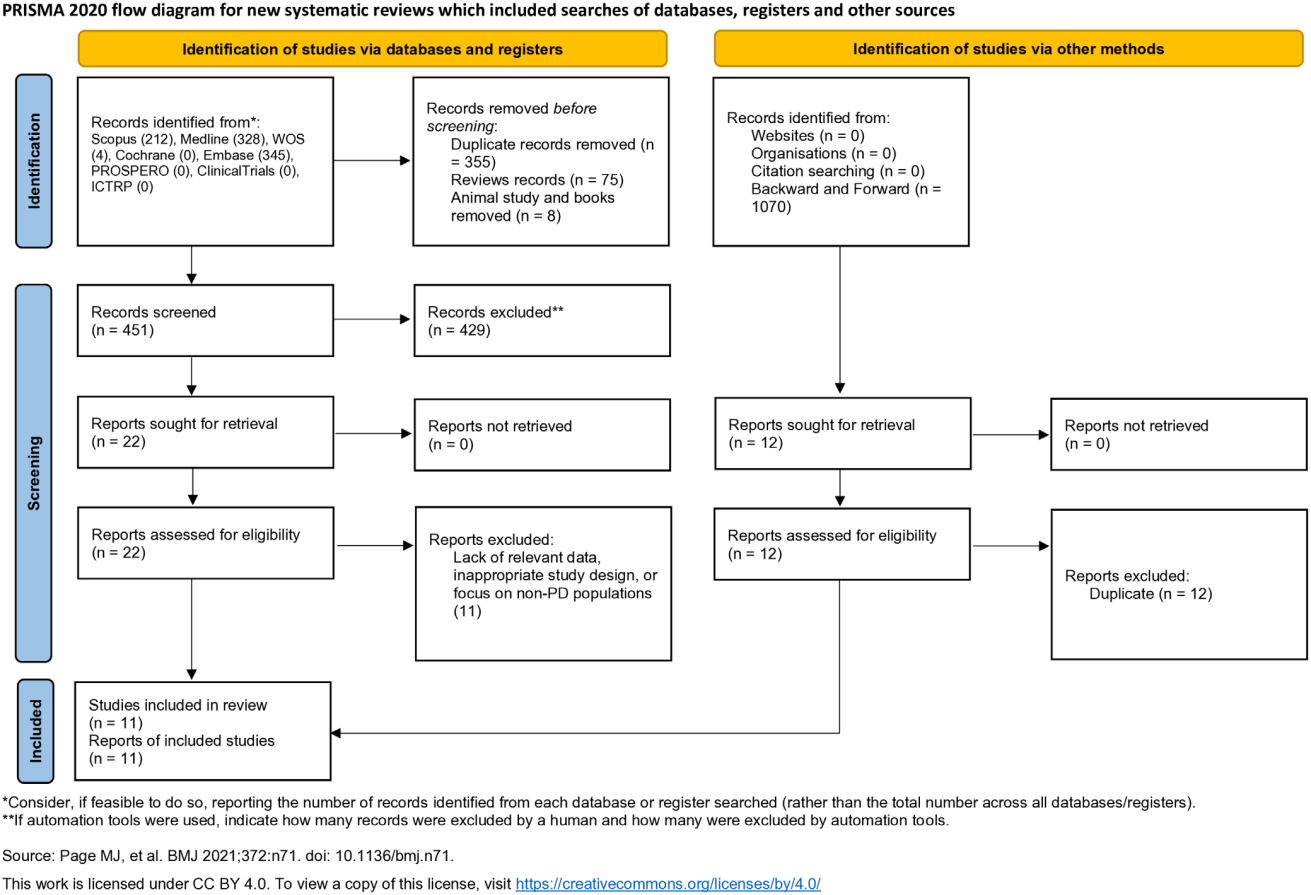
The PRISMA flow diagram for the study selection process.

The study selection process is illustrated in the PRISMA flow diagram (Figure 1). A total of 889 studies were initially identified through database searches, with 438 duplicates, reviews records, animal studies, and books subsequently removed. The titles and abstracts of the remaining 451 articles were screened by two researchers, leading to the selection of 22 full‐text studies for review. Of these, 11 full texts were unavailable, and 11 studies were excluded for various reasons [lack of relevant data, inappropriate study design, or focus on non‐PD populations (Figure 1)]. Ultimately, 11 studies were included in this meta‐analysis [1, 8, 9, 29, 30, 31, 32, 33, 34, 35, 36]. Furthermore, excluded studies were presented in the Data S1.

### 3.2 Study Characteristics

Published articles between 2017 and 2024 were included. The overall number of patients was 1462 [PD: 855 (58.48%), and non‐PD: 607 (41.52%)]. The records comprised a single cohort study [36], seven retrospective cross‐sectional studies [8, 9, 30, 32, 34, 35], and three prospective cross‐sectional studies [1, 8, 31, 33]. Furthermore, 10 studies were single center [8, 30, 31, 32, 33, 34, 35, 36], while another was multicenter [9]. Moreover, 10 studies were primary analyses [1, 8, 29, 30, 31, 32, 33, 34, 35, 36], and another was a secondary analysis [9] (Table 1).

**TABLE 1:** The summary of findings with the GRADE results for the primary outcome (the overall DTI‐ALPS index in PD‐HC comparison).

Author	Country	Year	Study design	Data	Type of center	Findings
Yao et al. (36)	China	2024	Cohort	Primary analysis	Single‐center	DTI‐ALPS index was significantly lower in PD patients compared to HC (*p* = 0.004), indicating glymphatic dysfunction. The DTI‐ALPS index negatively correlated with motor severity (UPDRS‐III scores) (*r* = −0.730, *p* < 0.001) and positively correlated with dopaminergic activity (SUVR) in the striatum, including mean putaminal SUVR (*r* = 0.560, *p* = 0.007) and mean caudal SUVR (*r* = 0.459, *p* = 0.032), suggesting its potential as a biomarker for disease severity and progression detection in PD
Wang et al. (1)	China	2024	Cross‐sectional	Primary analysis	Single‐center	The ALPS index was significantly lower in PD patients (*p* = 0.04), particularly in the PDD subgroup (*p* = 0.02), compared to HC. This suggests that reduced glymphatic function, as indicated by a lower ALPS index, may be associated with cognitive impairment in PD
Meng et al. (34)	China	2024	Cross‐Sectional	Primary Analysis	Single‐Center	The DTI‐ALPS index was significantly lower in PD patients compared to HC (left hemisphere: *p* = 0.0071; right hemisphere: *p* = 0.0221), especially in the medium to late‐stage group (left: *p* = 0.0164; right: *p* = 0.0379), indicating impaired glymphatic function as the disease progresses. The EPVS numbers were significantly higher in PD patients (all regions: *p* < 0.0001), particularly in the BG region (PDa: *p* = 0.0002; PDb: *p* < 0.0001), which correlated with motor symptom severity (UPDRS II: *r* = 0.4883, *p* = 0.0003; UPDRS III: *r* = 0.4390, *p* = 0.0013)
Qin et al. (9)	China	2023	Cross‐sectional	Secondary analysis	Multi‐center	The DTI‐ALPS index was significantly lower in PD patients than in HC (*p* = 0.031), indicating glymphatic dysfunction. The index was negatively correlated with motor symptom severity (MDS‐UPDRS‐III score) (*r* = −0.213, *p* = 0.008) and rigidity (*r* = −0.177, *p* = 0.029), suggesting its potential as a biomarker for PD motor symptoms
Bae et al. (8)	Republic of Korea	2023	Cross‐sectional	Primary analysis	Single‐center	The ALPS index was significantly lower in the PD group compared to HC (*p* < 0.001), suggesting glymphatic dysfunction. The index negatively correlated with motor severity (UPDRS‐III score) (*r* = −0.526, *p* < 0.001) and positively with cognitive function (MMSE: *r* = 0.377, *p* = 0.005; MoCA: *r* = 0.382, *p* = 0.004)
Bae et al. (29)	Republic of Korea	2023	Prospective cross‐sectional	Primary analysis	Single‐center	The ALPS index was significantly lower in the RBD and PD groups compared to HC (RBD vs HC: *p* = 0.001; PD vs HC: *p* < 0.001). A lower ALPS index was associated with a higher risk of conversion to α‐synucleinopathy (hazard ratio = 0.57 per 0.1 increase, *p* = 0.03), suggesting that impaired glymphatic function may be a predictor for the development of neurodegenerative disorders in RBD patients
Cai et al. (30)	China	2023	Cross‐sectional	Primary analysis	Single‐center	The ALPS index was significantly lower in both PD age groups (younger: *p* < 0.001; older: *p* < 0.001) compared to HC, indicating impaired glymphatic function in PD patients
Gu et al. (32)	China	2023	Cross‐sectional	Primary analysis	Single‐center	The ALPS index was significantly lower in PD patients compared to ET patients (*p* < 0.001) and HC (*p* < 0.001), indicating glymphatic dysfunction. There was a negative relation between the ALPS index and disease severity scores (UPDRS III) in PD patients (*r* = −0.180, *p* = 0.045), suggesting the ALPS index as a potential marker for assessing disease severity and progression in PD
Si et al. (35)	China	2022	Cross‐sectional	Primary analysis	Single‐center	The ALPS index was lower in the PD group compared to the piRBD (*p* = 0.036) and HC groups (PD vs HC: *p* < 0.001; piRBD vs HC: *p* = 0.001). The ALPS index negatively correlated with disease severity scores in both the piRBD (RBDQ‐HK II: *r* = −0.236, *p* = 0.010, corrected *p* = 0.045) and PD groups (PD‐EDS: *r* = −0.370, *p* = 0.019, corrected *p* = 0.045; PD‐sRBD: *r* = −0.431, *p* = 0.020, corrected *p* = 0.045; PD‐CI: *r* = −0.303, *p* = 0.007, corrected *p* = 0.045), suggesting its potential as a biomarker for assessing disease progression and severity
Ma et al. (33)	China	2021	Prospective cross‐sectional	Primary analysis	Single‐center	The ALPS index was significantly lower in the late‐stage PD group compared to the HC group (*p* = 0.006). In the early‐stage PD group, the ALPS index positively correlated with cognitive function (MMSE score) (β = 0.021, *p* = 0.029) and negatively with the EPVS score (β = −0.050, *p* = 0.034). In the late‐stage PD group, the ALPS index was inversely associated with age (β = −0.012, *p* = 0.004)
Chen et al. (31)	Taiwan	2021	Prospective cross‐sectional	Benchmark	Single‐center	The DTI‐ALPS index was significantly lower in the PD‐MCI (*p* = 0.012) and PDD (*p* < 0.001) groups compared to HC. The ALPS index was inversely correlated with plasma DNA levels (nuclear DNA: *r* = −0.278, *p* = 0.001; mitochondrial DNA: *r* = −0.201, *p* = 0.026), indicating that oxidative stress may play a role in glymphatic dysfunction in PD patients. There was a positive relation between the ALPS index and cognitive function (MMSE: *r* = 0.222, *p* = 0.013; CASI: *r* = 0.178, *p* = 0.046), suggesting that the glymphatic system might be associated with cognitive decline in PD

GRADE																
	Summary estimate					Heterogeneity		Grade								GRADE score
	Number of Studies	Number of participants	MD (95% CI)	SMD (95% CI)	P_SMD_	*I* ^2^	P_Q_	Downgrades					Upgrades			
								Risk of bias	Inconsistency	Indirectness	Imprecision	Publication bias	Effect size	Dose response	Adjusted for confounders	
**The overall ALPS index (PD‐HC)**	10	1361	−0.10 (−0.13, −0.08)	−0.61 (−0.72, −0.50)	***< 0.001***	0.00	0.69	□	□	□	□	□	□	□	□	**⊕⊕⊕⊕ High**

Abbreviations: ALPS, along the perivascular space; DTI, diffusion tensor imaging; DTI‐APS, diffusion tensor imaging along the perivascular space; EPVS, enlarged perivascular spaces; ET, essential tremor; HC, healthy control; MCI, mild cognitive impairment; MDS, Movement Disorders Society; MMSE, Mini‐Mental State Examination; MoCA, Montreal Cognitive Assessment; PD, Parkinson's Disease; PDD, Parkinson's Disease with dementia; piRBD, possible idiopathic rapid eye movement sleep behavior disorder; RBD, rapid eye movement sleep behavior disorder; SUVR, standardized uptake value ratio; UPDRS, Unified Parkinson's Disease Rating Scale.

### 3.3 Results of Syntheses

#### 3.3.1 The Overall ALPS‐Index

**FIGURE 2:**
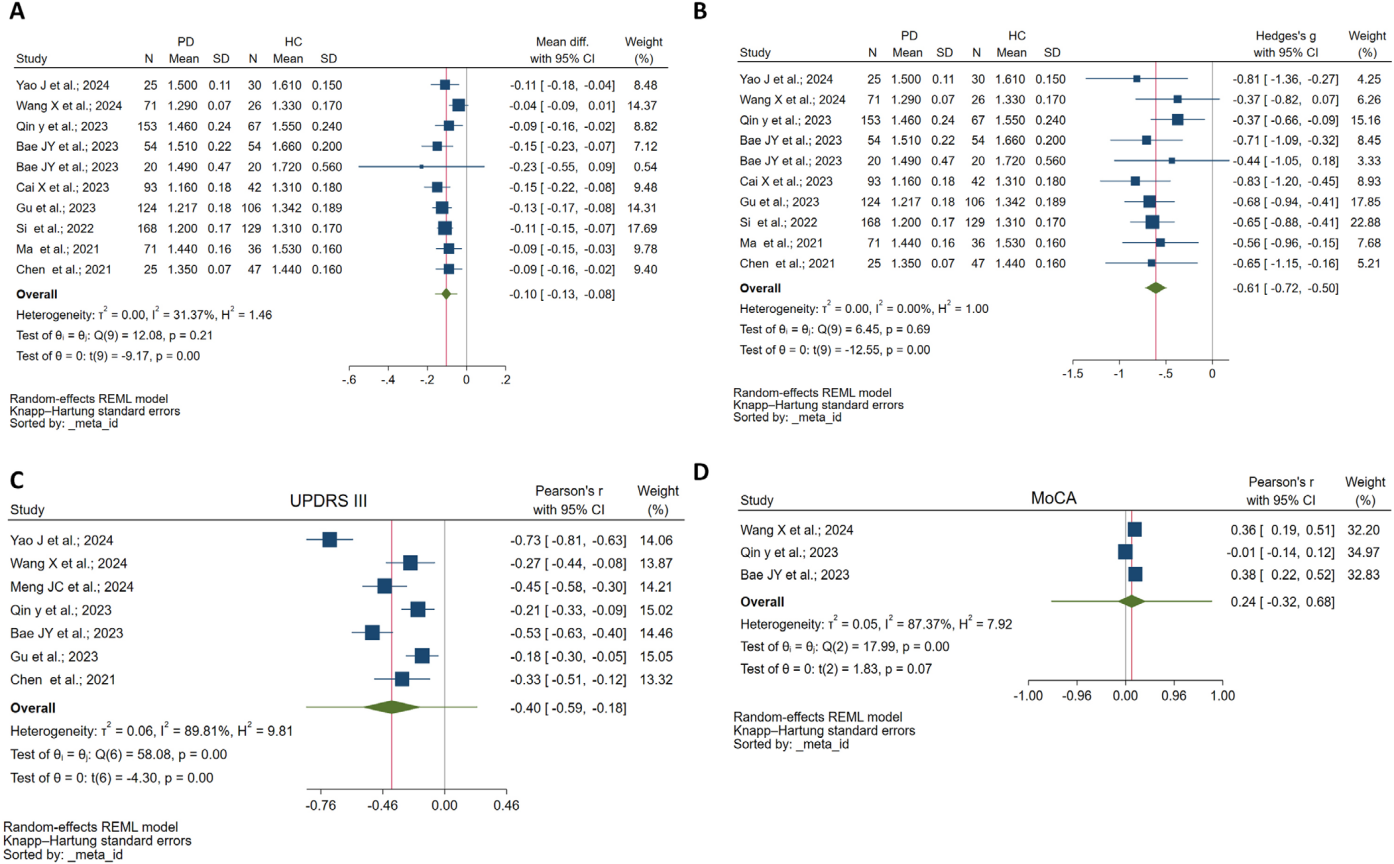
Meta‐analysis of the overall ALPS index and its relation with cognitive functions in Parkinson's disease (PD) and healthy control (HC) groups: (A) Forest plot comparing the overall ALPS index between PD and HC groups, showing a significant difference (*p* < 0.001). (B) Prediction interval for future studies on the overall ALPS index (−0.720, −0.497). (C) Correlation between the overall ALPS index and UPDRS III, demonstrating a significant association (*p* = 0.01). (D) Forest plot showing the correlation between the overall ALPS index and MoCA. A weak and statistically non‐significant correlation was observed (*r* = 0.24, 95% CI: −0.32 to 0.68, *p* = 0.07), with considerable heterogeneity across studies (*I* ^2^ = 87.37, *p* < 0.001 for heterogeneity).

Finally, 10 studies were conducted to compare the overall ALPS index between PD groups [1, 8, 9, 30, 31, 32, 33, 35, 36]. The MD was −0.10 (95% CI: −0.13, −0.08) (Figure 2A). This study shows that there was a significant difference between the PD and healthy control (HC) groups (*p* < 0.001) with −0.61 SMD (95% CI: −0.72, −0.50, *I* ^2^: 0.00, *p* = 0.69). Moreover, the prediction interval for the future study was (−0.720, −0.497) (Figure 2B).

#### 3.3.2 The Correlation Between the Overall ALPS Index and Cognitive Functions

Seven studies were conducted to compare the correlation between the overall ALPS index and UPDRS III [1, 8, 9, 31, 32, 34, 36]. Pearson's correlation analysis (r = −0.40, (95% CI: −0.59, −0.18, *I* ^2^: 89.81, *p* < 0.001)) revealed a statistically significant negative relationship (*p* < 0.001) (Figure 2C).

Three studies were conducted to compare the correlation between the overall ALPS index and MoCA [1, 8, 9]. Pearson's correlation analysis showed a weak and statistically non‐significant relationship between the ALPS index and MoCA (*r* = 0.24, 95% CI: −0.32 to 0.68, *p* = 0.07), with high heterogeneity among studies (*I* ^2^ = 87.37, *p* < 0.001 for heterogeneity) (Figure 2D).

### 3.4 Small Study Effect (Publication Bias)

**FIGURE 3:**
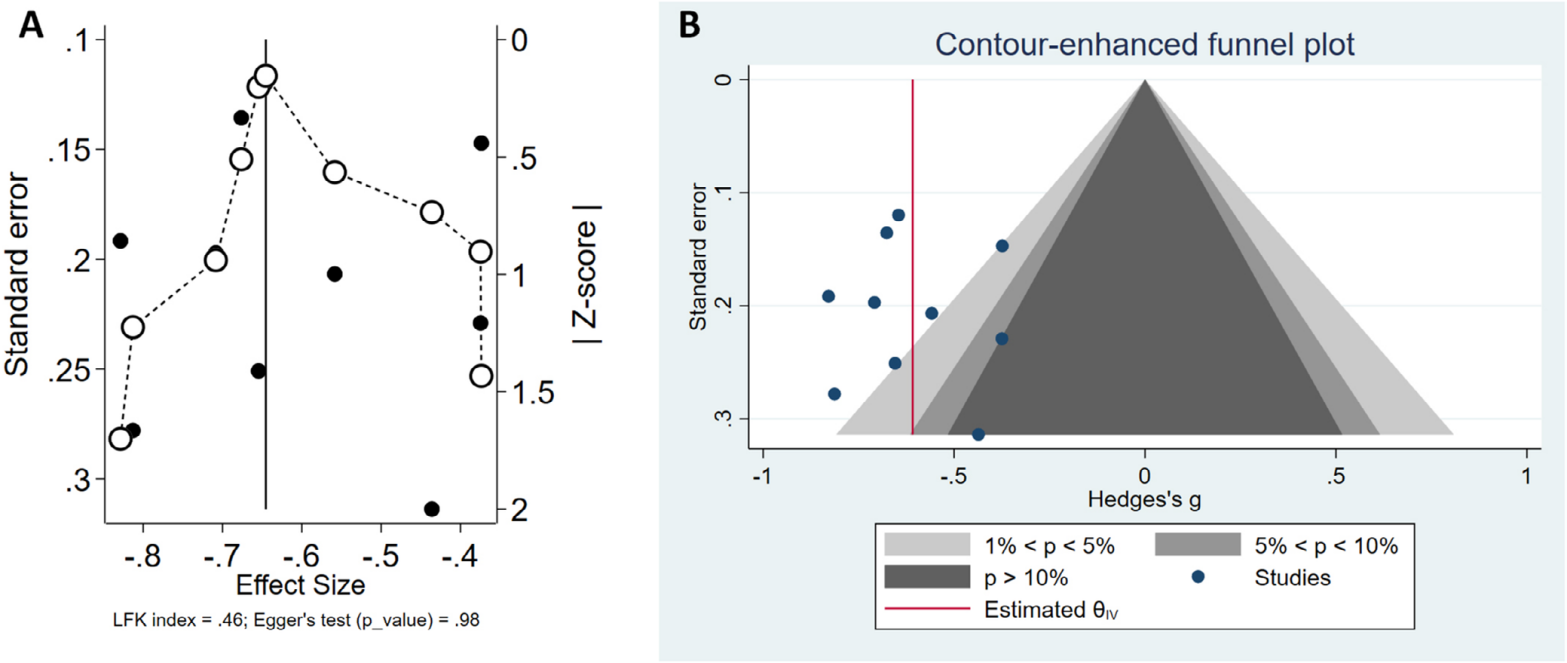
Publication bias and symmetry analysis for the overall ALPS‐index in PD and HC groups: (A) Doi plot showing no asymmetry (LFK = 0.46) between PD and HC groups. (B) Counter funnel plot indicating the absence of substantial publication bias for the overall ALPS index.

The initial analysis showed no considerable publication bias for the overall ALPS index and UPDRS III; however, Egger's test showed a possible publication bias in the correlation between the overall ALPS index and MoCA. Moreover, the trim and fill analysis did not suggest the missing studies for all variables (Data S1). However, the possibility of publication bias cannot be ignored. Moreover, the Doi plot showed that there is no asymmetry (LFK = 0.46) for the overall ALPS index between the PD‐HC group (Figure 3A) which is demonstrated in the counter funnel plot (Figure 3B). Moreover, the sensitivity analysis was presented in the Data S1.

### 3.5 Certainty of Evidence

The GRADE assessment for the primary outcome is shown in Table 1. The overall ALPS index in the PD‐HC comparison was highly graded.

## 4 Discussion

This systematic review and meta‐analysis investigated the potential of the DTI‐ALPS index in PD diagnosis, yielding promising results. Significant differences were observed between PD and healthy control groups regarding the overall ALPS index with medium effect size (SMD: −0.61, 95% CI: −0.72, −0.50, *p* = 0.69) indicating altered perivascular space integrity in PD. Additionally, a significant correlation was observed between the overall ALPS index and UPDRS III (r = −0.40, (95% CI: −0.59, −0.18, *I* ^2^: 89.81, *p* < 0.001)), suggesting a link between perivascular space changes and motor symptoms. However, no significant correlation was found with MoCA. Initial analysis indicated no considerable publication bias for the overall ALPS index and UPDRS III, but Egger's test suggested possible bias in correlation with MoCA. The GRADE assessment yielded high certainty for the primary outcome (overall ALPS index in PD‐HC comparison).

Potential biases indicated by Egger's test may stem from limited study sizes, selective reporting of positive findings, and the novelty of the ALPS index as a biomarker, which could influence study designs and reporting preferences. The small number of studies and underrepresentation of negative results may have affected the meta‐analysis. Egger et al. [24] discuss how publication bias can distort systematic review findings, particularly as studies with significant results are more likely to be published. Furthermore, methodological heterogeneity, such as differing imaging protocols and participant characteristics, can exacerbate variability. Higgins et al. [22] emphasize that these differences in study design and execution can introduce bias in meta‐analyses. To address these issues, future research should standardize imaging protocols, conduct larger multicenter studies, and report negative findings comprehensively. Tackling these biases will enhance the reliability and applicability of conclusions about the ALPS index as a PD biomarker.

The Bayesian meta‐analysis yielded a Log10 (BF10) of 30, indicating strong evidence for the presence of the overall DTI‐ALPS index in PD. This finding suggests that the observed association is statistically significant and supports the alternative hypothesis [37]. Although the current study was a classical systematic review and meta‐analysis that has a different methodological manner for the analysis of the data, this study showed that the overall ALPS index altered with perivascular space integrity in PD patients.

The reviewed literature highlights the DTI‐ALPS index as a potential biomarker for glymphatic dysfunction in PD, reinforcing its potential for monitoring disease progression and contributing to the understanding of PD pathophysiology [36, 38]. Notably, reduced glymphatic activity was observed in PD patients, with significantly lower Dzzassoc (diffusivity along the Z‐axis in the association fiber) and ALPS index values indicating impaired perivascular glymphatic function compared to healthy controls. Additionally, PD patients exhibited increased cerebrospinal fluid volumes. Importantly, a correlation emerged between glymphatic dysfunction (Dzzassoc and ALPS index) and disease severity, as measured by UPDRS‐III scores [36]. Furthermore, PD patients showed impaired perivascular glymphatic function (lower Dxxscr (diffusivity along the x‐axis in the superior corona radiata) and ALPS index) versus matched controls. Glymphatic metrics correlated significantly with motor and cognitive scores, indicating a link between glymphatic dysfunction and PD severity [8]. Longitudinal analyses have shown that lower DTI‐ALPS index values correlate with cognitive decline and disease severity, reinforcing its relevance as a potential biomarker for monitoring cognitive impairment in PD [1]. Although causality remains unclear, these findings suggest that the DTI‐ALPS index may serve as a marker for PD progression. Furthermore, enhancing glymphatic clearance may offer therapeutic potential for altering disease trajectory, warranting further exploration [39]. These findings underscore the critical role of the glymphatic system in PD pathophysiology and suggest the ALPS index's potential as a biomarker for evaluating cognitive decline and disease progression.

The DTI‐ALPS index and enlarged perivascular space (EPVS) measurements, particularly in the basal ganglia region, correlate strongly with age and PD progression. These metrics offer valuable non‐invasive tools for assessing glymphatic dysfunction and evaluating therapeutic interventions in clinical settings [34].

Glymphatic dysfunction is associated with cognitive impairment, potentially contributing to the accumulation of toxic proteins [40], which are thought to play a role in neuroinflammation and neurodegeneration. Specifically, PD is marked by misfolded α‐synuclein accumulation in Lewy bodies [41]. These findings highlight the association between glymphatic dysfunction, protein aggregation, and neurodegenerative diseases, emphasizing the need for further exploration of shared pathological mechanisms [42]. The glymphatic system plays a vital role in clearing solutes and transporting water within the central nervous system (CNS). Therefore, glymphatic system impairment facilitates waste accumulation, contributing to proteinopathies. Impaired brain fluid homeostasis results from several key mechanisms, including dilated or increased perivascular spaces, blood–brain barrier disruption, and compromised aquaporin‐4 (AQP‐4) water channels that regulate flow from periarterial spaces to brain parenchyma [43]. AQP‐4 dysfunction plays a pivotal role in glymphatic impairment, as demonstrated by animal models where AQP‐4 deletion exacerbates cognitive decline, beta‐amyloid accumulation, and alpha‐synuclein aggregation [44]. The interaction of the glymphatic system with various neurodegenerative mechanisms suggests AQP‐4 as a promising target for research and therapeutic interventions in PD, warranting further investigation into its role in neuroinflammation and glymphatic dysfunction [45].

The relationship between the DTI‐ALPS index and glymphatic function in humans remains a topic of ongoing debate. While the DTI‐ALPS index provides a promising non‐invasive biomarker for assessing perivascular space integrity, its direct representation of glymphatic activity has not been conclusively established. Pathophysiological studies are limited, and current evidence is largely correlative. Some studies suggest that the ALPS index reflects glymphatic system dysfunction due to its association with cerebrospinal and interstitial fluid exchange. However, the lack of direct mechanistic studies in humans leaves uncertainty regarding the extent to which the DTI‐ALPS index accurately quantifies glymphatic function. Confounding factors, such as AQP‐4 water channel functionality and blood–brain barrier integrity, may also influence the index, complicating its interpretation. Future research should prioritize longitudinal studies and experimental designs that explicitly explore the mechanistic link between DTI‐ALPS and glymphatic processes.

### 4.1 Study Limitations and Implications for Future Research

While prior studies have investigated the DTI‐ALPS index in neurodegenerative disorders, meta‐analyses specifically evaluating its diagnostic and prognostic value in PD remain limited. Our study systematically integrates recent findings, refines methodological approaches, and highlights research gaps to advance the understanding and clinical application of glymphatic dysfunction in PD. Although this systematic review and meta‐analysis provide a comprehensive overview of how the DTI‐ALPS index may be integrated into clinical practice, including early diagnosis, disease monitoring, and therapeutic efficacy assessment, certain limitations must be acknowledged.

Despite no missing studies suggested by trim & fill analysis, potential publication bias and small study effects may still influence the observed correlations between the ALPS index and MoCA. Additionally, high heterogeneity (*I* ^2^ values) and a limited number of studies on cognitive function introduce variability. The small sample size further affects generalizability and increases the risk of Type II errors, highlighting the need for larger, high‐quality studies. To identify potential sources of heterogeneity, we conducted subgroup analyses based on study characteristics and sensitivity analyses using multiple strategies, including the leave‐one‐out method and subgrouping by NOS quality assessment scores, study design, and type of center. However, substantial variability remained, likely due to differences in cognitive assessment methodologies, disease severity, and imaging protocols among studies. Future research should address these factors by adopting standardized cognitive evaluation methods and longitudinal research designs.

To assess the potential impact of study quality on our results, we conducted a sensitivity analysis by excluding studies with an NOS score of ≤ 6. The findings remained consistent, suggesting that the overall conclusions are robust. However, the inclusion of lower quality studies remains a limitation, and future research should prioritize high‐quality longitudinal studies to strengthen the evidence base for the DTI‐ALPS index as a biomarker for PD.

Additionally, a key limitation of this study is the absence of comparator groups such as multiple system atrophy (MSA), progressive supranuclear palsy (PSP), and dementia with Lewy bodies (DLB), making it unclear whether the glymphatic dysfunction observed in PD is unique or part of a broader neurodegenerative process. Glymphatic impairment has been reported in Alzheimer's disease (AD) and other neurodegenerative conditions [46], suggesting it may not be a disease‐specific hallmark. However, variations in dysfunction patterns across disorders may influence its diagnostic and prognostic value in PD. Future research should conduct direct comparative analyses, particularly involving AD, MSA, and amyotrophic lateral sclerosis (ALS), to determine whether glymphatic alterations in PD are distinct.

Single‐center studies in our meta‐analysis may introduce biases related to regional population characteristics, imaging protocols, and scanner variability. While subgroup analyses assessed inter‐study differences, future research should prioritize multicenter collaborations to enhance generalizability and reduce methodological discrepancies. Furthermore, our analysis is based on reported summary statistics from the reviewed records, assuming their validity. However, it is important to note that statistical analysis in the current study relies on the normality of data distributions, which was not explicitly reported in some included records. Also, causal inference limitations prevent establishing a direct causal relationship between glymphatic dysfunction and cognitive decline. Addressing these limitations through future research, such as longitudinal studies, mechanistic investigations, standardized protocols, and causal inference analyses, will strengthen our understanding of glymphatic function in PD.

These findings reinforce the pivotal role of glymphatic dysfunction in PD progression and support the DTI‐ALPS index as a potential biomarker for cognitive decline. Glymphatic impairment, influenced by AQP4 water channels, presents a promising target for therapeutic interventions. Clinically, early detection and glymphatic‐targeting therapies could improve patient outcomes. Despite this limitation, our findings provide strong support for the clinical applicability of the DTI‐ALPS index as a non‐invasive biomarker for assessing disease progression and potentially guiding therapeutic interventions in PD. Future research should investigate glymphatic function across multiple neurological disorders, elucidate the underlying mechanisms of glymphatic dysfunction, and develop novel therapeutic strategies. The translational implications of this research extend to improved diagnosis and treatment of PD, a deeper understanding of neurodegenerative diseases, and potential applications in other conditions associated with glymphatic dysfunction.

## 5 Conclusion

This systematic review and meta‐analysis study highlights the promising role of the DTI‐ALPS index as a noninvasive biomarker for PD diagnosis and progression monitoring. The findings demonstrate significant differences in ALPS index values between PD patients and healthy controls, as well as relations with disease severity, suggesting its potential utility in early diagnosis and tracking disease progression. However, the study also underscores limitations, including methodological variability, small sample sizes, and the lack of longitudinal and mechanistic studies necessary to establish causality. While the DTI‐ALPS index offers insights into glymphatic dysfunction, further research is required to refine its clinical application and address gaps in understanding its relationship with PD pathology. By consolidating current evidence, this study provides a foundation for future research aimed at standardizing imaging protocols, exploring causal mechanisms, and integrating the DTI‐ALPS index into clinical practice. These efforts could pave the way for more effective diagnostic and therapeutic strategies, ultimately improving outcomes for patients with PD.

## Disclosure

*Transparency statement*: The corresponding author Marziyeh Tahmasbi affirms that this manuscript is an honest, accurate, and transparent account of the study being reported; that no important aspects of the study have been omitted; and that any discrepancies from the study as planned (and, if relevant, registered) have been explained. The corresponding author Marziyeh Tahmasbi had full access to all of the data in this study and takes complete responsibility for the integrity of the data and the accuracy of the data analysis.

## Ethics Statement

The authors have nothing to report.

## Consent

The authors have nothing to report.

## Conflicts of Interest

All authors have read and verified the final version of the manuscript. The authors declare no conflicts of interest.

## Supporting information


Data S1.

## Data Availability

The data that supports the findings of this study are available in the Data S1 of this article.
