# Supplementary material for: Diffusion Tensor Imaging Along the Perivascular Space Is a Promising Imaging Method in Parkinson's Disease: A Systematic Review and Meta‐Analysis Study
Source: CNS Neurosci Ther. 2025 May 16;31(5):e70434. doi: 10.1111/cns.70434 (PMC12082281; doi:10.1111/cns.70434)
Supplement: Supplementary file 1 — Data S1. [file CNS-31-e70434-s001.docx]

**Supplementary Materials**

**APPENDIX I: Search terms and strategies**

Search strategy for **PubMed/Medline (NLM)**

Last searched November 13, 2024

(((((((((((((((((("Nervous System Diseases"[TIAB]) OR "Central Nervous System Diseases"[TIAB]) OR "Brain Diseases"[TIAB]) OR "Basal Ganglia Diseases"[TIAB]) OR "Parkinsonian Disorders"[TIAB]) OR "Parkinson’s Disease"[TIAB]) OR "Dementia, Vascular"[TIAB]) OR "Movement Disorders"[TIAB]) OR "Cognitive Dysfunction"[TIAB]) AND "Diagnostic Techniques and Procedures"[TIAB]) OR "Diagnostic Imaging"[TIAB]) OR "Magnetic Resonance Imaging"[TIAB]) OR "Neuroimaging"[TIAB]) OR "Diffusion Magnetic Resonance Imaging"[TIAB]) OR "Diffusion Tensor Imaging"[TIAB]) OR "Analysis Along the Perivascular Space"[TIAB]) OR "ALPS Index"[TIAB]) AND "Cardiovascular System"[TIAB]) OR "Central Nervous System"[TIAB]) OR "Brain"[TIAB]) OR "Glymphatic System"[TIAB])

Document Types: Article

Search strategy for **WEB OF SCIENCE (WoS)**

Last searched November 13, 2024

TS=(((((((((((((((((("Nervous System Diseases") OR "Central Nervous System Diseases") OR "Brain Diseases") OR "Basal Ganglia Diseases") OR "Parkinsonian Disorders") OR "Parkinson’s Disease") OR "Dementia, Vascular") OR "Movement Disorders") OR "Cognitive Dysfunction") AND "Diagnostic Techniques and Procedures") OR "Diagnostic Imaging") OR "Magnetic Resonance Imaging") OR "Neuroimaging") OR "Diffusion Magnetic Resonance Imaging") OR "Diffusion Tensor Imaging") OR "Analysis Along the Perivascular Space") OR "ALPS Index") AND "Cardiovascular System") OR "Central Nervous System") OR "Brain") OR "Glymphatic System")

Document Types: Article

Search strategy for **Scopus**

Last searched November 13, 2024

TITLE-ABS-KEY(((((((((((((((((("Nervous System Diseases") OR "Central Nervous System Diseases") OR "Brain Diseases") OR "Basal Ganglia Diseases") OR "Parkinsonian Disorders") OR "Parkinson’s Disease") OR "Dementia, Vascular") OR "Movement Disorders") OR "Cognitive Dysfunction") AND "Diagnostic Techniques and Procedures") OR "Diagnostic Imaging") OR "Magnetic Resonance Imaging") OR "Neuroimaging") OR "Diffusion Magnetic Resonance Imaging") OR "Diffusion Tensor Imaging") OR "Analysis Along the Perivascular Space") OR "ALPS Index") AND "Cardiovascular System") OR "Central Nervous System") OR "Brain") OR "Glymphatic System")

Document Types: Article

Search strategy for **Embase®**

Last searched November 13, 2024

TITLE-ABS-KEY(((((((((((((((((("Nervous System Diseases") OR "Central Nervous System Diseases") OR "Brain Diseases") OR "Basal Ganglia Diseases") OR "Parkinsonian Disorders") OR "Parkinson’s Disease") OR "Dementia, Vascular") OR "Movement Disorders") OR "Cognitive Dysfunction") AND "Diagnostic Techniques and Procedures") OR "Diagnostic Imaging") OR "Magnetic Resonance Imaging") OR "Neuroimaging") OR "Diffusion Magnetic Resonance Imaging") OR "Diffusion Tensor Imaging") OR "Analysis Along the Perivascular Space") OR "ALPS Index") AND "Cardiovascular System") OR "Central Nervous System") OR "Brain") OR "Glymphatic System")

Document Types: Article

Search strategy for **Cochrane**

Last searched November 13, 2024

("Nervous System Diseases" OR "Central Nervous System Diseases" OR "Brain Diseases" OR "Basal Ganglia Diseases" OR "Parkinsonian Disorders" OR "Parkinson’s Disease" OR "Dementia, Vascular" OR "Movement Disorders" OR "Cognitive Dysfunction") AND ("Diagnostic Techniques and Procedures" OR "Diagnostic Imaging" OR "Magnetic Resonance Imaging" OR "Neuroimaging" OR "Diffusion Magnetic Resonance Imaging" OR "Diffusion Tensor Imaging" OR "Analysis Along the Perivascular Space" OR "ALPS Index") AND ("Cardiovascular System" OR "Central Nervous System" OR "Brain" OR "Glymphatic System")

Document Types: Article

**APPENDIX II: NOS quality risk-of-bias assessment tool for observational studies**

**eTable 1.** The Newcastle-Ottawa Scale (NOS) quality assessment of the included studies in this meta-analysis (details).

| **Study** | **Selection of case and controls** | | | | **Comparability of cases**  **and controls** | **Exposure** | | | **Total** |
| --- | --- | --- | --- | --- | --- | --- | --- | --- | --- |
|  | **Is the case**  **definition**  **adequate** | **Representativeness**  **of the cases** | **Selection of**  **Controls** | **Definition of Controls** | **Comparability of cases and**  **controls on the basis of the**  **design or analysis** | **Ascertainment**  **of exposure** | **Same method of**  **ascertainment for**  **cases and controls** | **Non-**  **Response**  **Rate** |  |
| Yao et al. 2024, China | ☆ | - | ☆ | ☆ | ☆ | ☆ | ☆ | - | 6 |
| Wang et al. 2024, China. | ☆ | - | ☆ | ☆ | ☆ | ☆ | ☆ | - | 6 |
| Meng et al. 2024, China. | ☆ | ☆ | ☆ | ☆ | ☆ | ☆ | ☆ | - | 7 |
| Qin et al. 2023, China. | ☆ | ☆ | ☆ | ☆ | ☆ | ☆ | ☆ | ☆ | 8 |
| Bae et al. 2023, republic of Korea. | ☆ | - | ☆ | ☆ | ☆ | ☆ | ☆ | - | 6 |
| Bae et al. 2023, republic of Korea. | ☆ | - | ☆ | ☆ | ☆ | ☆ | ☆ | ☆ | 7 |
| Cai et al.2023, China. | ☆ | - | ☆ | ☆ | ☆ | ☆ | ☆ | ☆ | 7 |
| Gu et al. 2023, China. | ☆ | - | ☆ | ☆ | ☆ | ☆ | ☆ | - | 6 |
| Si et al. 2022, China. | ☆ | ☆ | ☆ | ☆ | ☆ | ☆ | ☆ | - | 7 |
| Ma et al. 2021, China. | ☆ | ☆ | ☆ | ☆ | ☆ | ☆ | ☆ | - | 7 |
| Chen et al. 2021, Taiwan. | ☆ | - | ☆ | ☆ | ☆ | ☆ | ☆ | - | 6 |

**APPENDIX III: Excluded studies with the reasons.**

**eTable 2.** Excluded studies.

| ID | Reason | Additional information |
| --- | --- | --- |
| Costa, T., et al., 2024 (1) | Review | NA |
| Zhou, C., et al., 2024 (2) | Irrelevant population. | NA |
| Pang, H. et al., 2024 (3) | Irrelevant population. | NA |
| Wood, K. H., et al., 2024 (4) | Irrelevant population. | NA |
| Xiaodan Liu et al., 2024 (5) | Irrelevant population. | The study involved 213 healthy participants. It aimed to investigate the relationship between iron deposition and the function of the glymphatic system in the normal aging brain. |
| Peikun He et al., 2023 (6) | Irrelevant study design. | The study lacked a control group. |
| Shijiao Tian et al., 2023 (7) | Irrelevant study design. | The study lacked a control group. |
| Y Liu et al., 2023 (5) | Irrelevant population. | NA |
| Junjun Wang et al., 2023 (8) | Irrelevant population | The study included adults aged ≥40 years and free of known dementia or stroke. Participants with MRI contraindications, serious head injury, intracranial surgery, or cancer were excluded. |
| Adam M. Wright et al., 2023 (9) | Irrelevant population. | Participants were from the Indiana Alzheimer’s Disease Research Center (IADRC), focusing on early-stage Alzheimer’s disease and white matter alterations. |
| Ruan, X., et al., 2022 (10) | Irrelevant study design. | NA |
| McKnight, C. D., et al., 2021 (11) | Irrelevant study design. | NA |
| Ting Shen et al., 2021 (12) | ALPS index not mentioned. | NA |
| Wei Zhou et al., 2020 (13) | Irrelevant study design. | NA |

**APPENDIX IV: Publication bias assessment using Egger’s tests, Egger’s graphs, trim&fill plots, and the possibility of heterogeneity.**

**eTable 3.** Egger tests results to evaluate the possibility of small study effect among results.

| **Groups** | **z** | **P-value** |
| --- | --- | --- |
| The overall ALPS-index | 0.02 | 0.98 |
| UPDRS III | - 0.84 | 0.4 |
| MoCA | 4.14 | ***< 0.001*** |

**
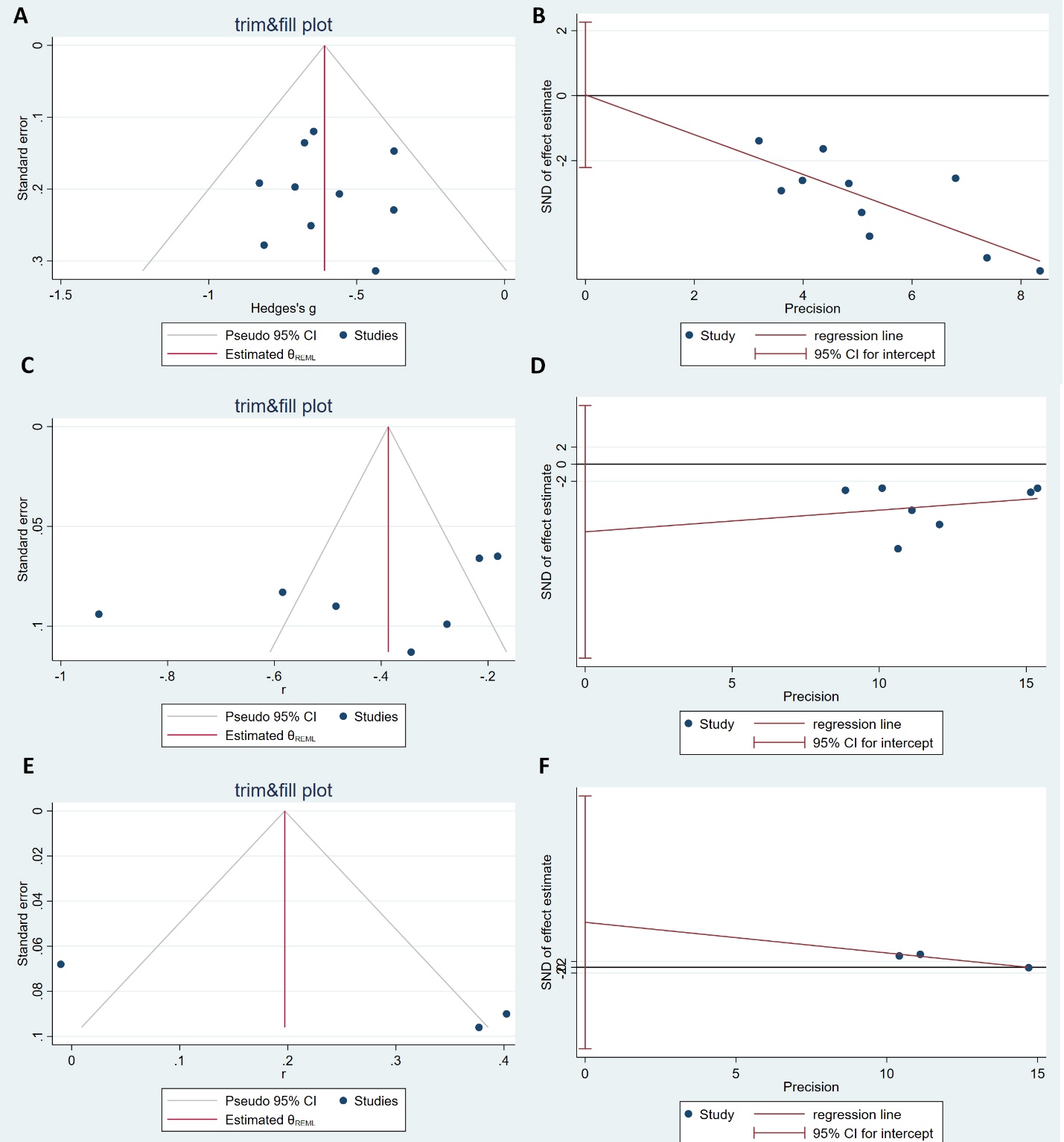
**

**eFigure 1.** Publication bias assessment using Egger’s graphs, and trim&fill plots. **A.** The trim&fill plot for left ALPS-index in PD-HC group, **B.** The Egger’s graph for the selected comparisons for correlation between ALPS-index and UPDRS III in PD-HC group, **C.** The trim&fill plot for correlation between ALPS-index and UPDRS III in PD-HC group, **D.** The Egger’s graph for the selected comparisons for correlation between ALPS-index and UPDRS III in PD-HC group, **E.** The trim&fill plot for correlation between ALPS-index and MoCA in PD-HC group, and **F.** The Egger’s graph for the selected comparisons for correlation between ALPS-index and MoCA in the PD-HC group.

**
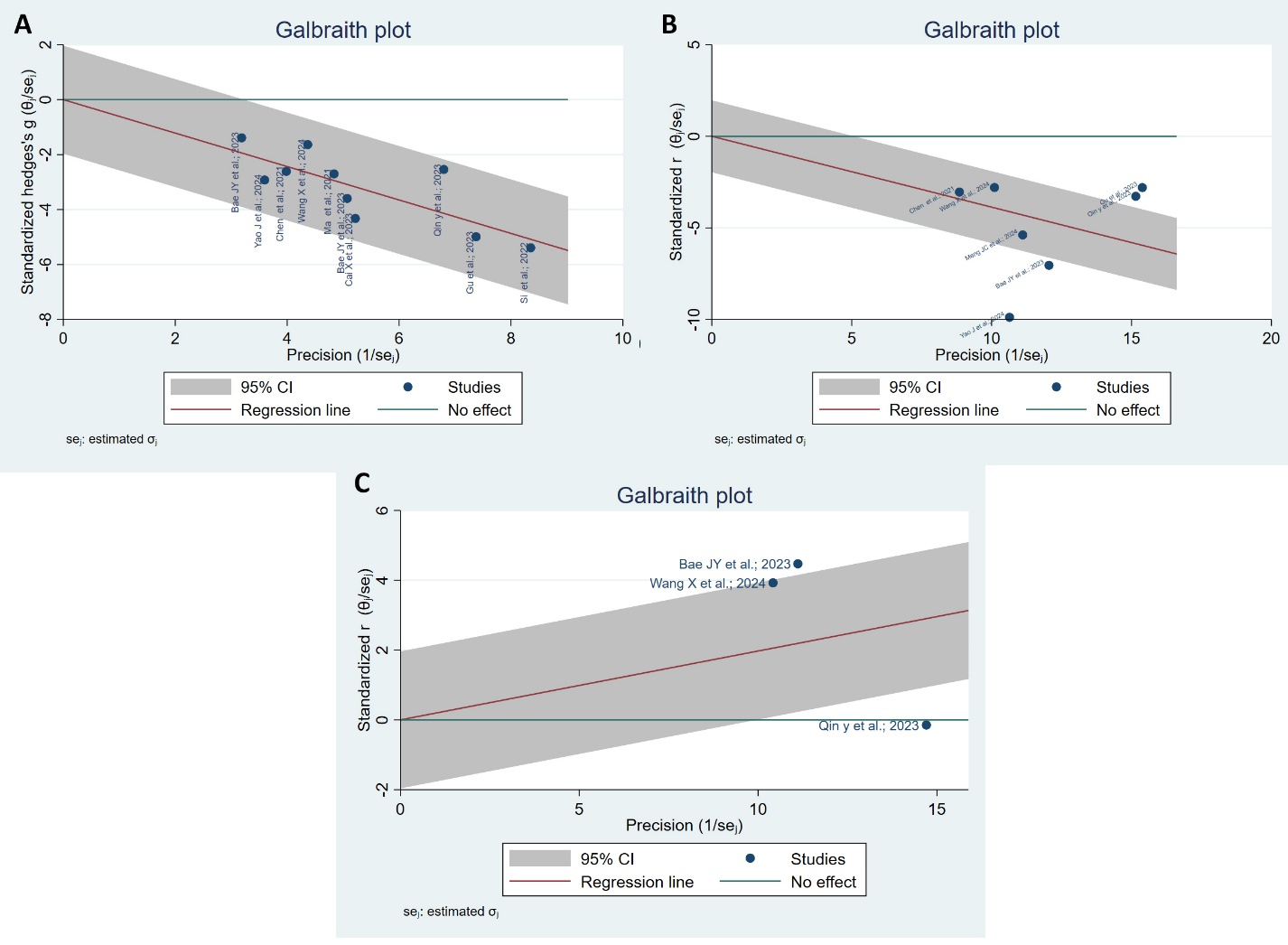
**

**eFigure 2.** Galbraith plot to assess the possibility of heterogeneity. **A.** The overall ALPS-index in PD-HC, **B.** correlation between the ALPS-index and MoCA in the PD-HC group, and **C.** correlation between ALPS-index and MoCA in the PD-HC group.

**APPENDIX V: Sensitivity analysis results**

Subgroup analysis by study quality: High-quality studies showed a stronger association.

**
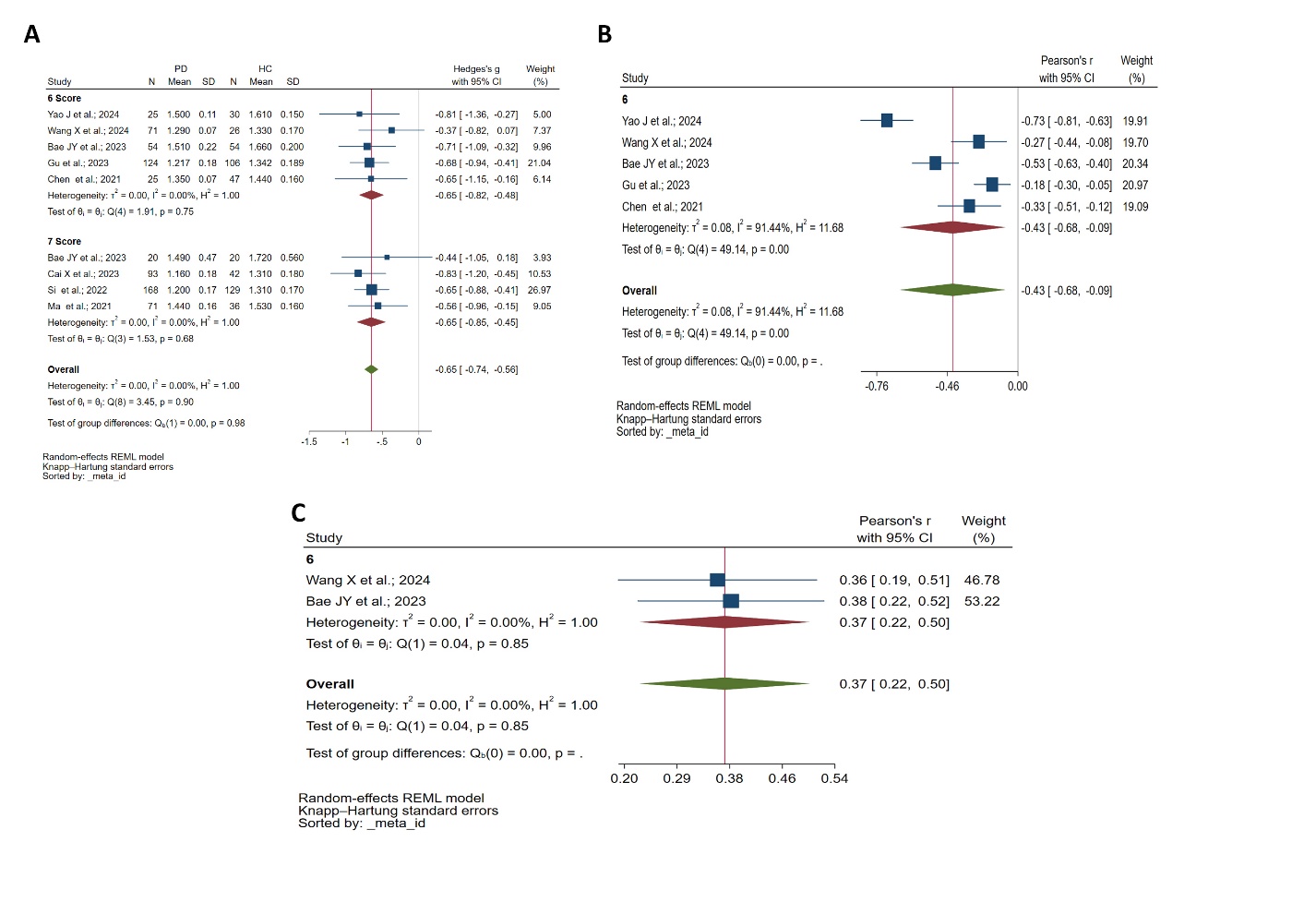
**

**eFigure 3.** Sensitivity analysis based on the NOS quality assessment categories. **A.** The subgroup based on the NOS quality assessment score for the overall ALPS-index in PD-HC group, **B.** The subgroup based on the NOS quality assessment score for UPDRS III, **C.** The subgroup based on the NOS quality assessment score for the MoCA.

Subgroup analysis by type of centers, study designs, and primary or secondary analysis: These analysis showed that there was no important factor to influence these results.

**
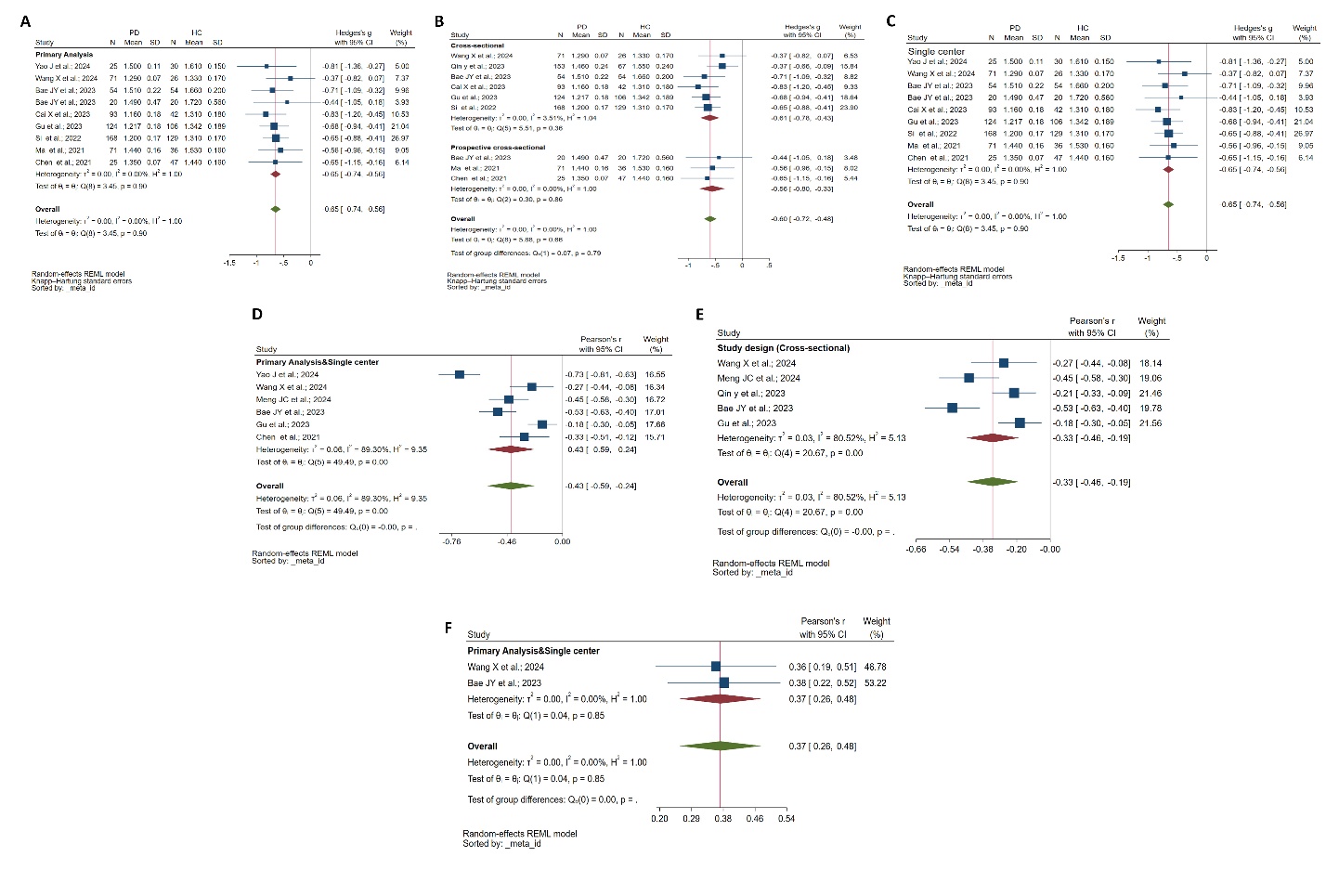
**

**eFigure 4.** Sensitivity analysis using the type of centers, study designs, and primary or secondary analysis. **A.** Sensitivity analysis using the primary or secondary analysis for the overall ALPS-index in PD-HC group, **B.** Sensitivity analysis using the study designs analysis for the overall ALPS-index in PD-HC group, **C.** Sensitivity analysis using the type of centers for the overall ALPS-index in PD-HC group, **D.** Sensitivity analysis using the primary or secondary and type of centers analysis for the UPDRS III, **E.** Sensitivity analysis using the study designs for the UPDRS III, **F.** Sensitivity analysis using the primary or secondary and type of centers analysis for the MoCA.

Leave-one-out analysis: The pooled effect size remained significant (p < 0.05) after removing each study individually for the overall ALPS-index, and UPDRS III. However, after excluding Qin y et al., 2023 (14) study the pooled Pearson’s r become significant.

**
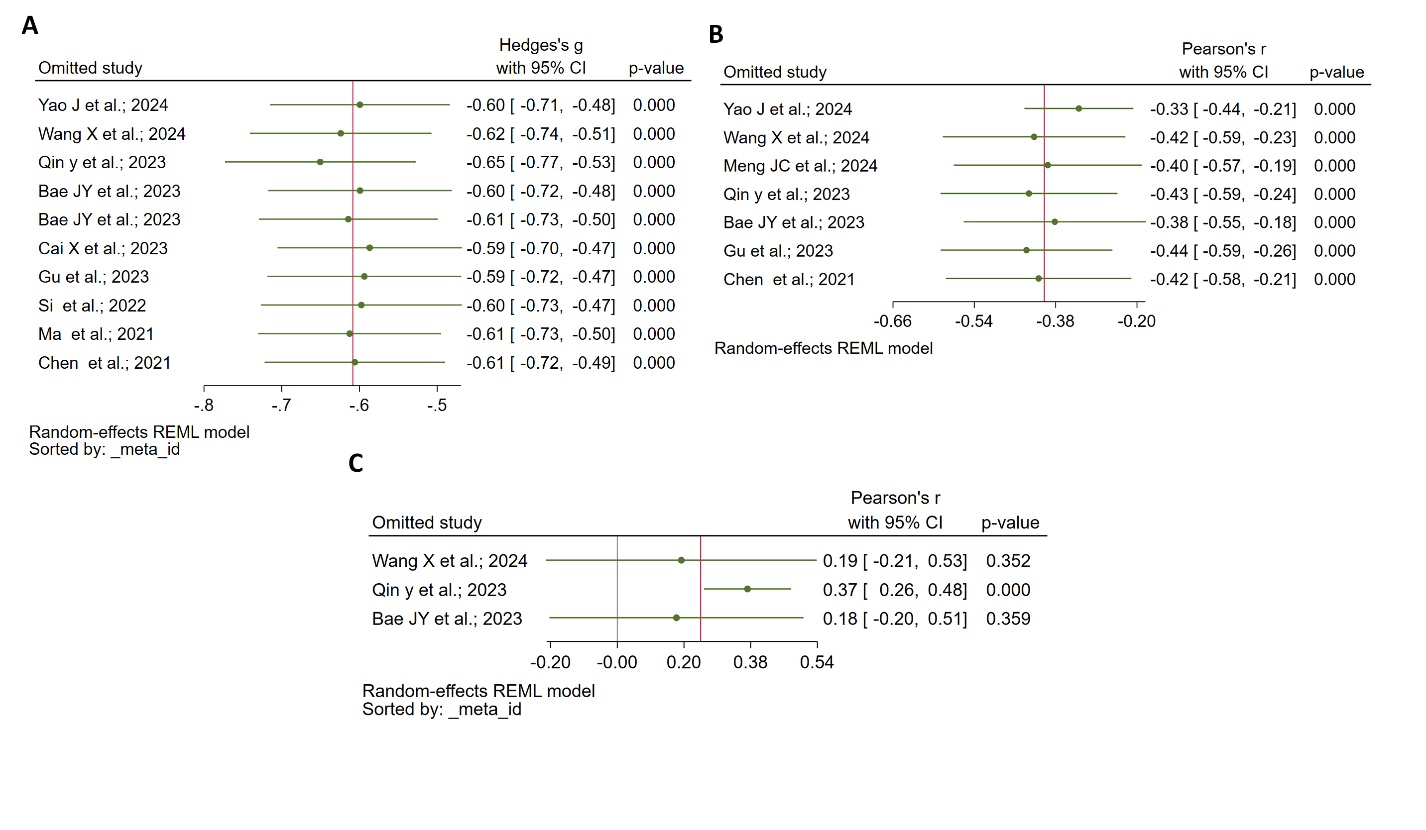
**

**eFigure 5.** Sensitivity analysis using the leave-one-out remove method. **A.** The leave-one-out remove method for the overall ALPS-index in the PD-HC group, **B.** Sensitivity analysis using the leave-one-out remove method for the UPDRS III, and **C.** Sensitivity analysis using the leave-one-out remove method for the MoCA.

**APPENDIX VI: The imaging protocols of the included articles.**

**eTable 4.** The imaging protocols of the included articles.

| **Parameters** | **Jung Bae et al. (15, 16)** | **Cai et al. (17)** | **Gu et al. (18)** | **Wang et al. (19)** | **Meng et al. (20)** | **Qin et al. (14)** | **Ma et al. (21)** | **Si et al. (22)** | **Chen et al. (23)** | **Yao et al. (24)** |
| --- | --- | --- | --- | --- | --- | --- | --- | --- | --- | --- |
| **MRI Scanner** | Philips Ingenia CX | GE Signa Excite HD | GE Discovery 750 | Ingenia Philips Healthcare | Siemens Skyra | Siemens TIM Trio | Philips Achieva TX | GE Discovery 750 | GE SIGNA | PET-MRI uPMR 790 |
| **Head Coil (channel)** | 32 | 8 | 8 | 15 | - | 12 | - | 8 | - | 32 |
| **Gradient Directions** | 32 | 25 | - | 48 | 30 | 64 | 31 | 30 | 13 | 32 |
| **TR (ms)** | 9900 | 8000 | 8000 | 8223 | 6600 | - | - | 8000 | 15800 | 4663 |
| **TE (ms)** | 77 | 76 | 80 | 85 | 94 | - | 93 | 80 | 77 | 78 |
| **FOV (mm²)** | 224×224 | 256×256 | 256×256 | 256×192 | 230×230 | - | - | 256×256 | 256×256 | 230×250 |
| **Voxel Size (mm³)** | 2×2×2 | 2×2×3 | - | 2×2×2 | 1.9×1.9×5 | 2×2×2 | - | - | 2×2×2.5 | 2×2×2 |
| **Slice Thickness (mm)** | 2 | 2.5 | 2 | 2 | 5 | - | 3 | 2 | 2.5 | 4 |
| **Matrix Size** | 112×112 | - | 128×128 | 128×96 | - | - | 128×128 | 128×128 | 118×128 |  |
| **Flip Angle** | 90° | 90° | 90° | - | - | 90° | - | 90° | - | 90° |
| **Software** | MATLAB | DTI Studio, MRIcroN | FSL | FSL | FSL | FSL | DTI Studio | FSL | dTV.II.13k+ | FSL |
| Note) DTI = Diffusion Tensor Imaging, MRI = Magnetic Resonance Imaging, TR = Repetition Time, TE = Echo Time, FOV = Field of View | | | | | | | | | | |

**References**

1. Costa T, Manuello J, Premi E, Mattioli I, Lasagna L, Lahoz CB, et al. Evaluating the robustness of DTI-ALPS in clinical context: a meta-analytic parallel on Alzheimer's and Parkinson's diseases. Sci Rep. 2024;14(1):26381.

2. Zhou C, Jiang X, Guan X, Guo T, Wu J, Wu H, et al. Glymphatic system dysfunction and risk of clinical milestones in patients with Parkinson disease. Eur J Neurol. 2024;31(12):e16521.

3. Pang H, Wang J, Yu Z, Yu H, Li X, Bu S, et al. Glymphatic function from diffusion-tensor MRI to predict conversion from mild cognitive impairment to dementia in Parkinson's disease. J Neurol. 2024;271(8):5598-609.

4. Wood KH, Nenert R, Miften AM, Kent GW, Sleyster M, Memon RA, et al. Diffusion Tensor Imaging-Along the Perivascular-Space Index Is Associated with Disease Progression in Parkinson's Disease. Mov Disord. 2024;39(9):1504-13.

5. Liu Y, Yuan J, Liao H, Tan C, Cai S. Glymphatic system functional changes of bilateral cerebral hemispheres in early-stage Parkinson disease patients complicated with unilateral limb motor symptoms. Chinese Journal of Medical Imaging Technology. 2023;39(12):1787-91.

6. He P, Shi L, Li Y, Duan Q, Qiu Y, Feng S, et al. The Association of the Glymphatic Function with Parkinson's Disease Symptoms: Neuroimaging Evidence from Longitudinal and Cross-Sectional Studies. Ann Neurol. 2023;94(4):672-83.

7. Tian S, Hong H, Luo X, Zeng Q, Huang P, Zhang M. Association between body mass index and glymphatic function using diffusion tensor image-along the perivascular space (DTI-ALPS) in patients with Parkinson's disease. Quant Imaging Med Surg. 2024;14(3):2296-308.

8. Wang J, Zhou Y, Zhang K, Ran W, Zhu X, Zhong W, et al. Glymphatic function plays a protective role in ageing-related cognitive decline. Age Ageing. 2023;52(7).

9. Wright AM, Wu YC, Chen NK, Wen Q. Exploring Radial Asymmetry in MR Diffusion Tensor Imaging and Its Impact on the Interpretation of Glymphatic Mechanisms. J Magn Reson Imaging. 2023.

10. Ruan X, Huang X, Li Y, Li E, Li M, Wei X. Diffusion Tensor Imaging Analysis Along the Perivascular Space Index in Primary Parkinson's Disease Patients With and Without Freezing of Gait. Neuroscience. 2022;506:51-7.

11. McKnight CD, Trujillo P, Lopez AM, Petersen K, Considine C, Lin YC, et al. Diffusion along perivascular spaces reveals evidence supportive of glymphatic function impairment in Parkinson disease. Parkinsonism Relat Disord. 2021;89:98-104.

12. Shen T, Yue Y, Ba F, He T, Tang X, Hu X, et al. Diffusion along perivascular spaces as marker for impairment of glymphatic system in Parkinson's disease. NPJ Parkinsons Dis. 2022;8(1):174.

13. Zhou W, Shen B, Shen WQ, Chen H, Zheng YF, Fei JJ. Dysfunction of the Glymphatic System Might Be Related to Iron Deposition in the Normal Aging Brain. Front Aging Neurosci. 2020;12:559603.

14. Qin Y, He R, Chen J, Zhou X, Zhou X, Liu Z, et al. Neuroimaging uncovers distinct relationships of glymphatic dysfunction and motor symptoms in Parkinson’s disease. Journal of Neurology. 2023;270(5):2649-58.

15. Bae YJ, Kim JM, Choi BS, Ryoo N, Song YS, Nam Y, et al. Altered Brain Glymphatic Flow at Diffusion-Tensor MRI in Rapid Eye Movement Sleep Behavior Disorder. Radiology. 2023;307(5).

16. Bae YJ, Kim JM, Choi BS, Choi JH, Ryoo N, Song YS, et al. Glymphatic function assessment in Parkinson's disease using diffusion tensor image analysis along the perivascular space. Parkinsonism and Related Disorders. 2023;114.

17. Cai X, Chen Z, He C, Zhang P, Nie K, Qiu Y, et al. Diffusion along perivascular spaces provides evidence interlinking compromised glymphatic function with aging in Parkinson's disease. CNS Neuroscience and Therapeutics. 2023;29(1):111-21.

18. Gu L, Dai S, Guo T, Si X, Lv D, Wang Z, et al. Noninvasive neuroimaging provides evidence for deterioration of the glymphatic system in Parkinson's disease relative to essential tremor. Parkinsonism and Related Disorders. 2023;107.

19. Wang X, Huang P, Haacke EM, Wu P, Zhang X, Zhang H, et al. MRI index of glymphatic system mediates the influence of locus coeruleus on cognition in Parkinson's disease. Parkinsonism and Related Disorders. 2024;123.

20. Meng JC, Shen MQ, Lu YL, Feng HX, Chen XY, Xu DQ, et al. Correlation of glymphatic system abnormalities with Parkinson’s disease progression: a clinical study based on non-invasive fMRI. Journal of Neurology. 2024;271(1):457-71.

21. Ma X, Li S, Li C, Wang R, Chen M, Chen H, Su W. Diffusion Tensor Imaging Along the Perivascular Space Index in Different Stages of Parkinson’s Disease. Frontiers in Aging Neuroscience. 2021;13.

22. Si X, Guo T, Wang Z, Fang Y, Gu L, Cao L, et al. Neuroimaging evidence of glymphatic system dysfunction in possible REM sleep behavior disorder and Parkinson’s disease. npj Parkinson's Disease. 2022;8(1).

23. Chen HL, Chen PC, Lu CH, Tsai NW, Yu CC, Chou KH, et al. Associations among Cognitive Functions, Plasma DNA, and Diffusion Tensor Image along the Perivascular Space (DTI-ALPS) in Patients with Parkinson's Disease. Oxidative Medicine and Cellular Longevity. 2021;2021.

24. Yao J, Huang T, Tian Y, Zhao H, Li R, Yin X, et al. Early detection of dopaminergic dysfunction and glymphatic system impairment in Parkinson's disease. Parkinsonism and Related Disorders. 2024;127.
